# Singlet Oxygen Induced Products of Linoleates, 10- and 12-(Z,E)-Hydroxyoctadecadienoic Acids (HODE), Can Be Potential Biomarkers for Early Detection of Type 2 Diabetes

**DOI:** 10.1371/journal.pone.0063542

**Published:** 2013-05-15

**Authors:** Aya Umeno, Mototada Shichiri, Noriko Ishida, Yoshiko Hashimoto, Kaori Abe, Masatoshi Kataoka, Kohzoh Yoshino, Yoshihisa Hagihara, Nanako Aki, Makoto Funaki, Yasuhiko Asada, Yasukazu Yoshida

**Affiliations:** 1 Health Research Institute, National Institute of Advanced Industrial Science and Technology, Takamatsu, Kagawa, Japan; 2 Health Research Institute, National Institute of Advanced Industrial Science and Technology, Ikeda, Osaka, Japan; 3 Clinical Research Center for Diabetes, Tokushima University Hospital, Tokushima, Tokushima, Japan; 4 Depertment of Applied Biological Science, Faculty of Agriculture, Kagawa University, Kita-gun, Kagawa, Japan; Broad Institute of Harvard and MIT, United States of America

## Abstract

Current diagnostic tests such as glycemic indicators have limitations for early detection of impaired glucose tolerance (IGT), which leads to diabetes. Oxidative stress induced by various oxidants in a random and destructive manner is considered to play an important role in the pathophysiology of a number of human disorders and diseases such as impaired glucose tolerance. We have developed an improved method for the measurement of *in vivo* lipid peroxidation, where the presence of 8-iso-prostaglandin F_2α_ (8-iso-PGF_2α_), hydroxyoctadecadienoic acids (HODEs), hydroxyeicosatetraenoic acids (HETEs), and 7-hydroxycholesterol (7-OHCh), as well as their parent molecules, linoleic acid (LA) and cholesterol (Ch), was determined by performing LC-MS/MS (for 8-iso-PGF_2α_, HODE, and HETE) and GC-MS (for 7-OHCh, LA, and Ch) after reduction with triphenyl phosphine and saponification by potassium hydroxide. We then applied this method to volunteers (n = 57), including normal type (n = 43), “high-normal” (fasting plasma glucose, 100–109 mg/dL, n = 7), pre-diabetic type (IGT, n = 5), and diabetic type (n = 2) subjects who are diagnosed by performing oral glucose tolerance tests (OGTTs). Several biomarkers in plasma, such as insulin, leptin, adiponectin, interleukin-6, tumor necrosis factor-α, high sensitivity-C-reactive protein, HbA1c, and glucose levels were measured during OGTT. We found that the fasting levels of (10- and 12-(Z,E)- HODE)/LA increased significantly with increasing levels of HbA1c and glucose during OGTT and with insulin secretion and resistance index. In conclusion, 10- and 12-(Z,E)-HODE may be prominent biomarkers for the early detection of IGT and “high-normal” type without OGTT.

## Introduction

In 2011, there were about 366 million diabetes patients ages 20–79 years worldwide, but it was estimated that there will be 552 million diabetes patients worldwide within the next 20 years [International Diabetes Federation. Diabetes e-Atlas. Available at http://www.eatlas.idf.org. Accessed Aug. 6, 2012]. Diabetes is associated not only with increased coronary artery and vascular diseases, but also with blindness, amputations, and renal disease. It is necessary to recognize and treat this devastating disease early in its progression to postpone or even prevent the serious complications associated with diabetes. To diagnose diabetes by an oral glucose tolerance test (OGTT), the cut-off value for the blood glucose level between pre-diabetes and diabetes is either 126 mg/ml at fasting or 200 mg/ml at 120 min after administrating 75 g glucose; healthy individuals have fasting and 120 min OGTT concentrations of less than 110 and 140 mg/dL, respectively. The committee of the Japan Diabetic Society recommends that subjects with a fasting plasma glucose (FPG) value of 100–109 mg/dL be classified as “high-normal” in the normal range of glucose metabolism disorders and that subjects with a “high-normal” FPG values undergo a 75 g OGTT for diagnosis as normal-, pre-diabetic-, or diabetic- type [1]. It is very important to clarify the states of pre-diabetes, determined as both impaired glucose tolerance (IGT) and impaired fasting glycaemia (IFG), and “high-normal”. Furthermore, it is highly crucial to identify potent biomarkers that would enable us to determine glucose homeostasis disorder at its early stage. From this point of view, several traditional biomarkers have been proposed, such as malondialdehyde, catalase [2], thioredoxin [3], cholesterol oxides [4], cytokines, chemokines [5], nitric oxide (NO) metabolites [6], and hydroxybutyrate [7].

Oxidative stress is a common pathogenic factor that has been suggested to lead to insulin resistance, β-cell dysfunction, IGT, and IFG. Lipid peroxidation products have received considerable attention as indices for oxidative stress since lipids are the most susceptible to oxidation *in vivo*. Moreover, the biological significance of lipid oxidation products has been studied extensively. For this purpose, various products have been measured using diverse methods and techniques. However, lipid peroxidation yields numerous products, which makes it difficult to measure the extent of lipid peroxidation *in vivo*. F_2_-isoprostanes and isofurans, which consist of a series of chemically stable prostaglandin F_2_-like compounds formed by a mechanism independent of the cyclooxygenase (COX) pathway, have been widely assessed as oxidative stress markers *in vivo* [8]–[11]. Many researchers have reported the relevance of F_2_-isoprostanes as stress markers for pathogenesis, for example, urinary 8-iso-prostaglandin F_2α_ (8-iso-PGF_2α_) in smokers and nonsmokers [12], [13], isoprostanes in Alzheimer’s disease [14], and F_2_-isoprostanes in human atherosclerotic lesions [15], [16]. Other important oxidized lipids obtained from arachidonic acid are hydroxyeicosatetraenoic acids (HETEs), which are formed by enzymatic (cytochrome P450 and hydroxylase) [17], [18] and nonenzymatic pathways. Several isomers of HETEs have been reported *in vivo*, such as 5-, 8-, 9-, 12-, 15-, and 20-HETE, although the clinical significance of each isomer has yet to be elucidated.

Hydroxyoctadecadienoic acids (HODEs) [19]–[22] derived from linoleic acid (LA) have recently attracted attention, and some reports have described the detection of these molecules, for example, the formation of 9-hydroxy LA in erythrocyte membranes of diabetes patients [20] and hydroxy fatty acid in atherosclerotic patients [19]. Furthermore, the usefulness of HODE as a biomarker has been reported in recent literature [23]–[32]. HODEs formed by free-radical-mediated oxidation consist of 4 isomers, 13-hydroperoxy-9(Z), 11(E)-octadecadienoic acid (13-(Z,E)-HPODE), 13-hydroperoxy-9(E), 11(E)-octadecadienoic acid (13-(E,E)-HPODE), 9-hydroperoxy-10(E), 12(Z)-octadecadienoic acid (9-(E,Z)-HPODE), and 9-hydroperoxy-10(E), 12(E)-octadecadienoic acid (9-(E,E)-HPODE). On the other hand, singlet oxygen and ozone oxidize LAs by nonradical oxidation to form 13-hydroperoxy-9(Z), 11(E)-octadecadienoic acid (13-(Z,E)-HPODE), 10-hydroperoxy-8(E), 12(Z)-octadecadienoic acid (10-(E,Z)-HPODE), 12-hydroperoxy-9(Z), 13(E)-octadecadienoic acid (12-(Z,E)-HPODE), 9-hydroperoxy-10(E), 12(Z)-octadecadienoic acid (9-(E,Z)-HPODE). Thus, 10- and 12-(Z,E)-HPODEs are specific oxidation products for singlet oxygen [33]. It should be noted that the number of lipid peroxidation products formed in biological fluids and tissues depends on the rates of metabolism, clearance, and formation.

In this study, we focused on the detection of the IGT and “high-normal” states by using the lipid peroxidation biomarkers mentioned above, clarifying the pathogenic mechanisms promoting oxidative stress, and determining the method of early detection of the disease.

## Materials and Methods

### Ethics Statement

This study was approved by the institutional review boards of the National Institute of Advanced Industrial Science and Technology and by the ethics committees of the Tokushima University Hospital. All subjects gave their written informed consent after the purpose of this study was completely explained.

### Materials

8-iso-PGF_2α_, 8-iso-PGF_2α_-d_4_, 5-HETE, 12-HETE, 15-HETE, 13-hydroxy-9Z, 11E-octadecadienoic acid (13-(Z,E)-HODE), 9-(Z,E)-HODE, and 13-HODE-d_4_ were obtained from Cayman Chemical Company (MI, USA). 9-(E,E)-HODE, 13-(E,E)-HODE, 10-(Z,E)-HODE, and 12-(Z,E)-HODE were obtained from Larodan Fine Chemicals AB (Malmo, Sweden). 7β-OHCh were obtained from Steraloid Inc. (RI, USA), and their isotopes 7β-OHCh-d_7_ were obtained from Medical Isotopes Inc. (NH, USA). Other materials were of the highest grade commercially available.

### Subjects

**Figure 1:**
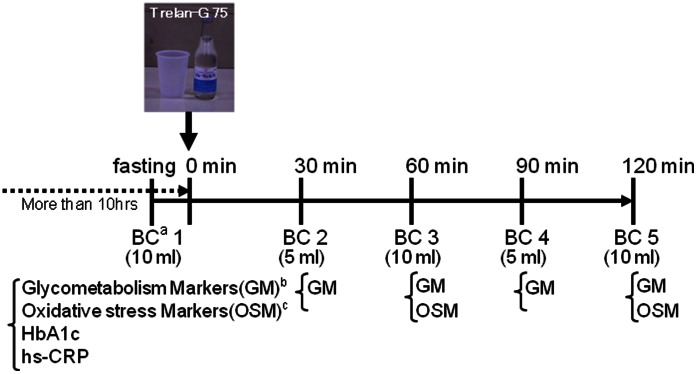
The study design of OGTT performed in this study. Abbreviations: a, Blood collection; b, Glycometabolism Markers (glucose, insulin, leptin, adiponectin, IL-6, TNF-α); c, Oxidative stress markers (9-(Z,E)-HODE, 9-(E,E)-HODE, 12-(Z,E)-HODE, 10-(Z,E)-HODE, 13-(Z,E)-HODE, 13-(E,E)-HODE, 5HETE, 12HETE, 15HETE, isoPs, linoleic acid, total cholesterol, 7β-hydroxycholesterol).

We studied 57 volunteers without any specific diseases. As shown in Fig. 1, an OGTT was performed for 2 h, collecting blood every 30 min in ethylenediaminetetraacetic acid disodium salt (EDTA–2 Na)-containing tubes. Plasma and erythrocytes were separated immediately after collection by centrifugation at 1500×g for 10 min at 4°C. Subsequently, plasma was frozen and stored at –80°C until analysis. For individuals whose fasting blood glucose levels exceeded 126 mg/ml OGTT was performed under a careful monitoring of their blood glucose levels by a diabetes specialist. No apparent adverse event was observed.

### Sample Processing

Plasma (200 µL) was mixed with 300 µL saline. Subsequently, 500 µL methanol containing the internal standards 8-iso-PGF_2α_-d_4_ (50 ng), 13-HODE-d_4_ (50 ng), 7β-OHCh-d_7_ (19 ng), and 100 µM butylated hydroxytoluene (BHT) was added to the samples. This was followed by the reduction of hydroperoxides using an excess amount of triphenylphosphine (final concentration, 1 mM) at room temperature for 30 min. Next, the reduced sample was mixed with 1 M KOH in methanol (500 µL) under nitrogen and incubated on a shaker for 30 min in the dark at 40°C. The mixture was cooled on ice and acidified with 10% acetic acid in water (2 mL) and extracted with chloroform and ethyl acetate (chloroform:ethyl acetate = 4∶1, v/v, 5 mL). The sample was mixed using a vortex mixer for 1 min and centrifuged at 1750×*g* for 10 min at 4°C. The chloroform and ethyl acetate layer was concentrated to around 1 mL after the removal of the water layer and divided equally into 2 portions.

### Analysis of t8-iso-PGF_2α_, HETE, and HODE by Liquid Chromatography-mass/mass (LC-MS/MS)

The divided chloroform and ethyl acetate solution was evaporated to dryness under nitrogen. The derivatized sample was reconstituted with methanol and water (methanol:water = 70∶30, v/v, 200 µL), and a portion of the sample (10 µL) was subjected to LC-MS/MS analysis. LC was carried out on an ODS column (Hypersil Gold, 3.0 µm, 100×2.1 mm, Thermo Fisher Scientific, CA, USA) in a column oven (CTO –20A, Shimadzu, Kyoto, Japan) set at 30°C. The LC apparatus consisted of an autosampler (SIL –20AC, Shimadzu, Kyoto Japan) and a pump (LC –20AB, Shimadzu, Kyoto Japan). The eluent condition was a gradient comprising solvent A, 2 mM ammonium acetate in water, and solvent B, methanol and acetonitrile (methanol:acetonitrile = 5∶95), at a flow rate of 0.2 mL/min. The initial composition of the gradient was 80% A and 20% B. It was folded for 2 min, and the composition was changed to 50% A and 50% B after 45 min. MS was carried out using a Thermo Finnigan TSQ Quantum Discovery Max, a triple-quadrupole mass spectrometer (Thermo Fisher Scientific, CA, USA) fitted with an electrospray ionization (ESI) source. ESI was carried out at a needle voltage of 4.2 kV. Nitrogen was used as the sheath gas (40 psi) and auxiliary gas (12 units). The capillary was heated to 270°C, and the mass spectrometers were optimized to achieve the maximum sensitivity. A specific precursor-to-product-ion transition was carried out by selected reaction monitoring (SRM) after collision-induced dissociation in the negative mode. Argon was used as the collision gas, and the collision cell pressure was 1.5 mTorr. The precursor, product ions, and collision energy were determined after the optimization of MS/MS as follows: m/z = 353.5 and 192.6–193.6 at 27 eV for 8-iso-PGF_2α_, m/z = 357.0 and 196.5–197.5 at 27 eV for 8-iso-PGF_2α_-d_4_, m/z = 319.0 and 114.5–115.5 at 10 eV for 5-HETE, m/z = 319.3 and 162.8–163.8 at 15 eV for 12-HETE, m/z = 319.3 and 202.5–203.5 at 10 eV for 15-HETE, m/z = 295.0 and 194.6–195.6 at 21 eV for both 13-(Z,E)-HODE and 13-(E,E)-HODE, m/z = 295.0 and 170.5–171.5 at 24 eV for both 9-(E,Z)-HODE and 9-(E,E)-HODE, m/z = 295.0 and 182.6–183.6 at 22 eV for both 10-(Z,E)-HODE and 12-(Z,E)-HODE, and m/z = 299.0 and 197.6–198.6 at 15 eV for 13-HODE-d_4_.

### Analysis of 7-OHCh, Ch, and LA by Gas Chromatography-mass (GC-MS)

The other portion of the chloroform and ethyl acetate solution was also evaporated to dryness under nitrogen. A silylating agent N,O-bis(trimethylsilyl)-trifluoroacetamide (BSTFA, 50 µL) was added to the dried residue. The solution was vigorously mixed by vortexing for 0.5 min and incubated for 60 min at 60°C to obtain trimethylsilyl esters and ethers. An aliquot of this sample was injected into a gas chromatograph (GC 6890 N, Agilent Technologies, Palo Alto, CA, USA) that was equipped with a quadrupole mass spectrometer (5973 Network, Agilent Technologies). A fused-silica capillary column (HP-5MS, 5% phenyl methyl siloxane, 30 m×0.25 mm, Agilent Technologies) was used. Helium was used as the carrier gas at a flow rate of 1.2 mL/min. Temperature programming was carried out from 60°C to 280°C at 10°C/min. The injector temperature was set at 250°C, and the temperatures of the transfer line to the mass detector and ion source were 250°C and 230°C, respectively. The electron energy was set at 70 eV. 7β-OHCh, Ch, and LA were identified on the basis of their retention times and mass patterns; ions having m/z = 456 for and 7β-OHCh, 458 for Ch, and 337 for LA were selected for the quantification. 7β-OHCh, Ch, and LA were identified quantitatively by using β-OHCh-d_7_ as internal standards.

### Other Parameters as Biomarkers

Glucose, insulin, leptin, adiponectin, interleukin (IL)-6, tumor necrosis factor (TNF)-α, and high sensitivity-C-reactive protein (hs-CRP) were estimated by enzyme-linked immunosorbent assay (ELISA) method with commercial kits. (HbA1c; RAPIDIA Auto HbA1c-L (TFB Inc.), Glucose; Cica liquid GLU J (KANTO CHEMICAL Co., Inc. ), Insulin; LUMIPULSE presto insulin (Fujirebio Inc.), Leptin; Human leptin RIA kit (Millipore Co., Inc.), Adiponectin; CircuLexTM Human adiponectin ELISA Kit CY-8050 (MBL), IL-6; Human IL-6 CLEIA Fujirebio (Fujirebio. Inc.), TNF-α; Quantikine HS ELISA Human TNF-α Immunoassay (R&D Syatems. Inc.), and hs-CRP; CircuLexTM Human HS-CRP ELISA Kit CY-8071 (MBL). Normal levels of markers were taken from information provided by the manufacturers (SRL, Inc. Tokyo, Japan, CycLex Co. Ltd. Nagano, Japan, and MBL Co. Ltd. Nagoya, Japan), as follows: HbA1c, 4.3%–5.8%; hs-CRP, 0.61–2.09 ng/mL; glucose, 70–109 mg/dL; leptin, male 0.9–13.0 ng/mL and female 2.5–21.8 ng/mL; adiponectin, >4.0 µg/mL; IL-6, <4.0 pg/mL; and TNF-α, 0.6–2.8 pg/mL (as fasting normal levels).

The homeostasis model assessment of insulin resistance (HOMA-IR) and Matsuda index have been proposed as indices for insulin resistance and homeostasis assessment. HOMA-IR is calculated by fasting plasma glucose (mg/dL)×fasting insulin/405 (normal level, <1.6; insulin resistance, >2.5; according to Japan Diabetes Society). The insulinogenic index (I.I.) was calculated as the ratio of the increase in insulin to the increase in plasma glucose at 30 min after the 75-g glucose load, (insulin 30 min–0 min)/(plasma glucose 30 min–0 min) (normal level, >0.4; according to Japan Diabetes Society). Matsuda index was calculated by plasma glucose and insulin concentrations during OGTT [34, http://mmatsuda.diabetes-smc.jp/english.html].

### Statistics

Statistical analyses were carried out on a Microsoft personal computer by using SPSS software version 14.0 (SPSS Inc., Chicago, IL, USA). One-factor repeated measures design analysis of variance (ANOVA) was used to examine the main effect of elapsed time from glucose injection on each index. Significant effects were followed by Tukey's HSD multiple comparisons. A *P*-value of less than 0.05 was considered significant. Correlations were also analyzed by the Pearson test performed using SPSS software version 14.0, and a *P*-value of less than 0.05 was considered significant. Data were expressed as the mean ± SD.

## Results

### Characteristics of Subjects in OGTT

**Figure 2:**
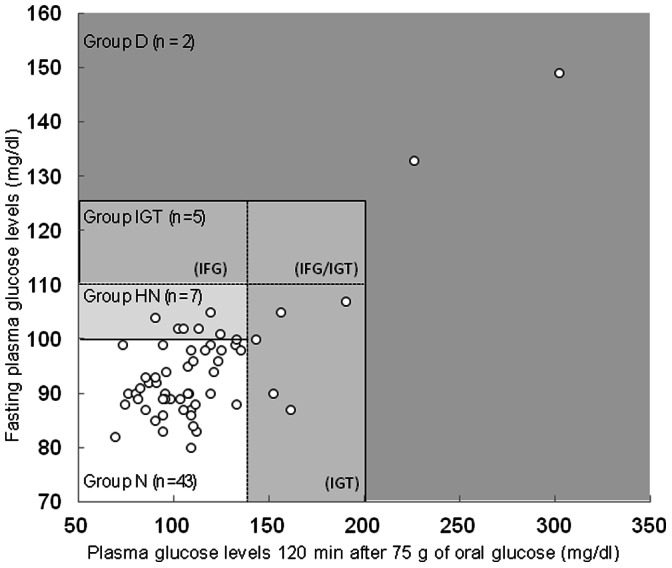
Classification of subjects by oral glucose tolerance test in this study. Group N, Normal-type; Group HN, High-Normal-type; Group IGT, Pre-diabetic-type; Group D, Diabetic-type.

We studied 57 volunteers who had not been diagnosed with any specific diagnoses of diabetes. The main characteristics and metabolic parameters of the subjects following 75 g OGTT are shown in Table 1 and Figure 2. As shown, 43 subjects were characterized as normal type (Group N), 7 as “high-normal” type (Group HN), and 5 as pre-diabetes (IGT, Group IGT). Unexpectedly, we found that there were 2 subjects with diabetic type (Group D). Body weight, body mass index (BMI), HbA1c, and hs-CRP levels tended to increase with deterioration of glucose tolerance. Glucose levels of Group HN were significantly higher (p<0.01) than those of Group N after fasting and 60 min after administration of glucose, while those at 120 min after glucose administration were not different from Group N. OGTT glucose levels of all subjects in Group IGT and Group D at 60 and 120 min after the administration were higher than 140 mg/dL. It is considered, in general, that postprandial glucose levels in Group N are less than 140 mg/dL and that the OGTT glucose levels of Group N at 120 min after the administration of glucose return to the fasting plasma glucose level. Some subjects showed insulin abnormalities in Groups HN, IGT, and D, as compared to the reference normal levels of 1.84–12.2 µU/mL (fasting plasma level). Levels of leptin, adiponectin, IL-6, and TNF-α were fairly constant during OGTT and within the normal reference levels.

**Table 1:** Characteristics of subjects at entry into the oral glucose tolerance test (OGTT).

		Group N	Group HN	Group IGT	Group D
		Normal-type	High-Normal -type^d^	Pre-Diabetes (IGT)	Diabetic type
*N*		43	7	5	2
Gender (M/F )		16/27	6/1	3/2	2/0
Age (years )		37.9±7.4	44.7±6.0	41.4±12.7	43.0
Height (cm )		163.3±8.4	170.8±5.7	164.7±9.5	173.0
Weight (kg )		60.5±11.2	69.7±10.0	69.1±12.6	83.0
BMI (kg/m^2^ )		22.6±3.6	23.8±2.4	25.3±1.9	27.7
HbA1c (% )		5.2±0.3	5.2±0.2	5.4±0.3	6.8
hs-CRP (µg/ml )		0.7±0.9	1.4±2.2	1.2±1.0	3.1
Glucose (mg/dl )	0^a^	90.8±5.1	102.3±1.7**	97.8±8.9*	141.0
	60^b^	122.6±29.9	175.7±39.2**	186.4±39.6**	259.5
	120^c^	101.3±16.9	112.3±14.5	160.4±17.8**	264.0
Insulin (µU/ml )	0	5.6±3.2	9.1±5.9	5.0±2.1	9.6
	60	55.2±36.0	78.4±37.9	51.2±30.7	49.0
	120	43.7±33.2	48.1±20.7	62.2±34.0	57.9
Leptin (ng/ml )	0	7.4±4.7	6.1±2.2	6.7±1.4	6.4
	60	7.3±4.5	5.8±2.0	6.2±1.1	5.6
	120	6.9±4.2	5.6±2.0	6.3±1.3	5.3
Adiponectin(µg/ml )	0	7.9±3.7	7.1±3.0	6.7±2.9	5.2
	60	7.7±3.7	6.5±2.6	6.8±3.3	4.3
	120	7.8±3.3	6.8±2.6	7.1±2.7	4.5
IL-6 (pg/ml )	0	2.2±1.6	2.7±1.4	2.4±0.6	2.4
	60	2.1±1.3	2.3±0.6	2.2±0.5	1.7
	120	2.3±1.8	2.2±0.7	2.2±1.0	1.7
TNF-α(pg/ml )	0	1.0±0.7	0.9±0.5	0.9±0.2	1.4
	60	0.9±0.7	0.8±0.5	0.8±0.2	1.5
	120	0.9±0.6	0.9±0.6	0.8±0.2	1.1
HOMA - IR		1.3±0.7	2.3±1.5*	1.2±0.6	3.4
L/A		1.2±1.0	1.0±0.2	1.3±0.9	1.2
Insulinogenic Index		1.8±1.5	1.0±0.9	0.6±0.3	ND
Matsuda index 5		7.8±3.8	3.9±1.4	6.4±2.6	ND
Matsuda index 3		9.1±5.2	4.5±1.7	7.0±4.0	2.8

Data are presented as mean ± standard deviation. One-factor completely randomized design analysis of variance (ANOVA) was used to examine the main effect of subject groups on each index. Significant effects were followed by Tukey's HSD multiple comparisons.

* p<0.05,

** p<0.01 compared with Group N. a, fasting; b, 60 min after 75 g of oral glucose; c, 120 min after 75 g of oral glucose; d, fasting sugar, 100 -109 mg/dl (defined by the Japan Diabetes Society) Abbreviations : BMI, body mass index; HOMA-IR, homeostasis model assessment of insulin resistance; L/A, Leptin/Adiponectin; hs-CRP, high sensitivity C - reactive protein; ND data not determined.

### Correlations of 10- and 12-(Z,E)-HODE with HbA1c and Glucose

**Figure 3:**
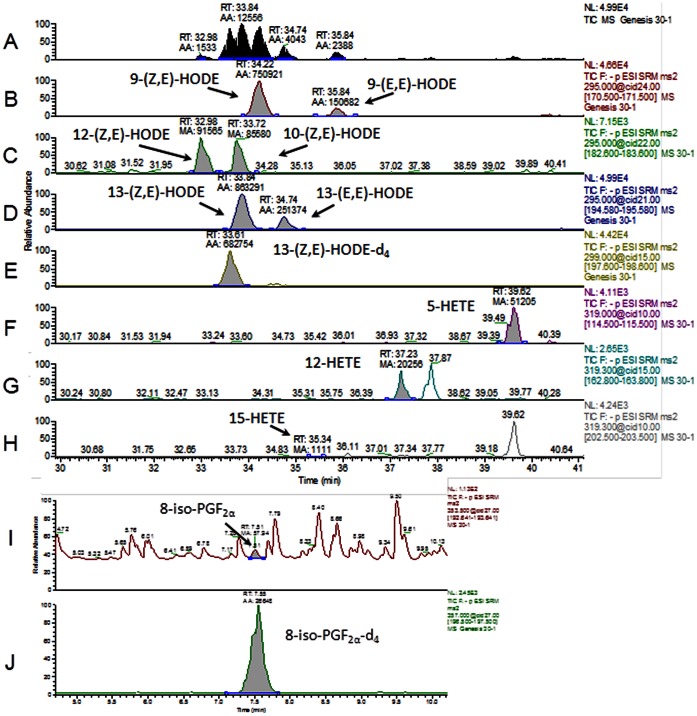
Typical chromatograms obtained from LC-MS/MS of human plasma samples measured in this study. (a) Total ion chromatogram, (b) 9-(Z,E)- and (E,E)-HODE, (c) 10- and 12-(Z,E)-HODE, (d) 13-(Z,E)- and (E,E)-HODE, (e) 13-(Z,E)-HODE-d_4_, (f) 5-HETE, (g) 12-HETE, (h) 15-HETE, (i) 8–iso-PGF_2α_, and (j) 8-iso-PGF_2α_-d_4._

**Figure 4:**
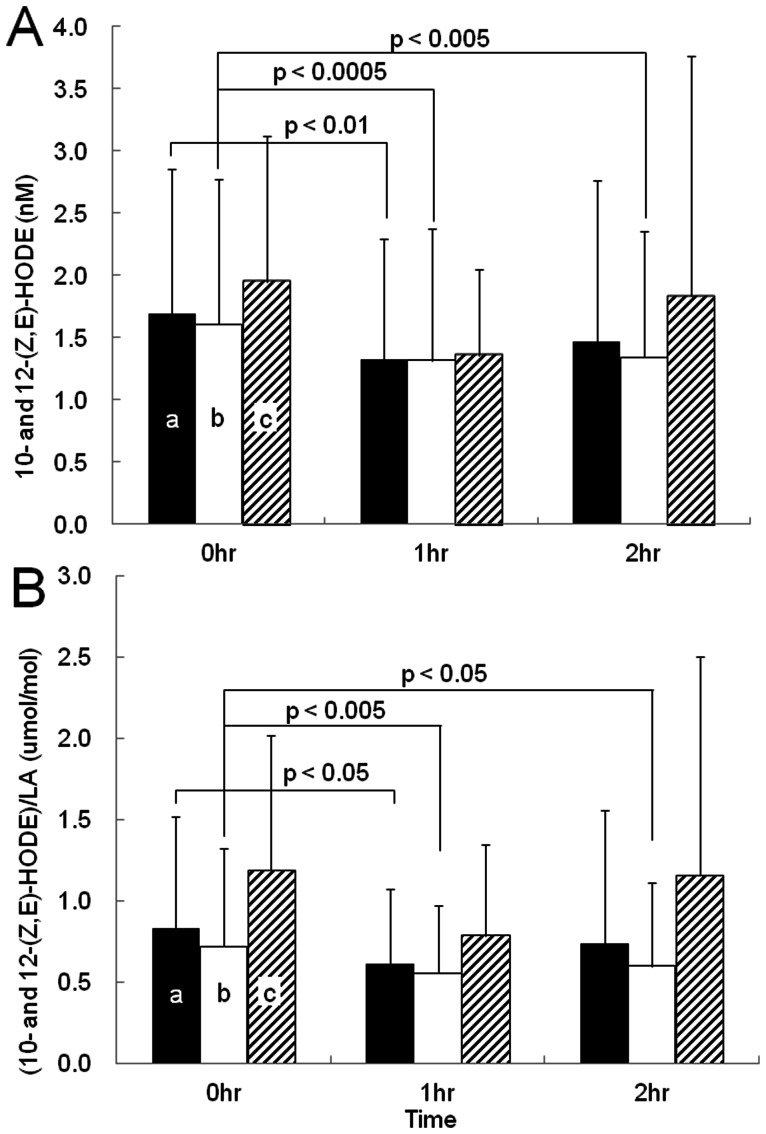
Plasma levels of 10- and 12-(Z,E)-HODE and those divided by linoleic acid (LA) during OGTT. One-factor repeated measures design analysis of variance (ANOVA) was used to examine the main effect of elapsed time from glucose injection on each index. Significant effects were followed by Tukey's HSD multiple comparisons. a, All subjects; b, Group N; c, Group HN, Group IGT, and Group D.

We measured several oxidative stress markers in plasma, including HODE, HETE, and 8-iso-PGF_2α._ Typical chromatograms from LC-MS/MS are shown in Figure 3. 9- and 13-(Z,E)–HODE, 9- and 13-(E,E)-HODE, 10- and 12-(Z,E)-HODE, 5-, 12-, 15-HETE, and 8-iso-PGF_2α_ were separated clearly on the chromatogram, and their quantitative values were obtained by comparison with their internal standards (13-(Z,E)-HODE-d_4_ and 8-iso-PGF_2α_-d_4_; Fig. 3). As shown in Table 2, among many oxidative stress markers studied, 10- and12-(Z,E)-HODE correlated significantly with HbA1c, glucose, and insulin during OGTT. 10- and12-(Z,E)-HODE levels and those divided by LA, parent molecular of HODEs, during OGTT are shown in Figure 4A and B, respectively. 10- and 12-(Z,E)-HODE (A) and (10- and 12-(Z,E)-HODE)/LA (B) levels in the plasma of all subjects (a) was significantly reduced 60 min after the administration of glucose (*p*<0.01, *p*<0.05, respectively). Moreover, these levels for Group N (b) were significantly reduced at 60 and 120 min after the administration of glucose, compared to the fasting level (*p*<0.0005–0.05). On the other hand, these levels in Groups HN, IGT, and D (c) were not significantly different for individuals during OGTT. Additionally, the levels of LA did not fluctuate during OGTT (data not shown).

**Table 2:** Correlation between the fasting plasma levels of lipid oxidation stress markers and glycometabolism markers, insulin secretion, and insulin resistance during OGTT.

	0 min											60 min						120 min						Index		
	Glucose	Insulin	Leptin	Adipo nectin		IL-6	TNF-α	HbA1c	CRP	HOMA - IR	L/A	Glucose	Insulin	Leptin	Adipo nectin	IL-6	TNF-α	Glucose	Insulin	Leptin	Adipo nectin	IL-6	TNF-α	Insulinogenic index	Matsuda index 5point	Matsuda index 3point
HbA1c	**	(–)	(–)		(–)	(–)	(–)	ND	**	*	(–)	**	(–)	(–)	(–)	(–)	*	**	(–)	(–)	(–)	(–)	(–)	(–)	(–)	(–)
9-(Z,E)-HODE/LA	(–)	(–)	(–)		(–)	(–)	(–)	(–)	(–)	(–)	(–)	**	(–)	(–)	(–)	(–)	(–)	(–)	(–)	(–)	(–)	(–)	(–)	#	(–)	(–)
9-(E,E)-HODE/LA	(–)	*	(–)		(–)	(–)	(–)	(–)	(–)	**	(–)	(–)	(–)	(–)	(–)	(–)	(–)	(–)	(–)	(–)	(–)	(–)	(–)	(–)	(–)	(–)
10-(Z,E)-HODE/LA	**	(–)	(–)		(–)	(–)	(–)	*	(–)	(–)	(–)	**	(–)	(–)	(–)	(–)	(–)	**	(–)	#	(–)	(–)	(–)	#	(–)	(–)
12-(Z,E)-HODE/LA	**	(–)	(–)		(–)	(–)	(–)	*	*	(–)	(–)	**	*	(–)	(–)	(–)	(–)	*	*	(–)	(–)	(–)	(–)	(–)	(–)	#
**(10- and 12-(Z,E)-HODE/LA**	**	**(–)**	**(–)**		**(–)**	**(–)**	**(–)**	*	*	**(–)**	**(–)**	**	**(–)**	**(–)**	**(–)**	**(–)**	**(–)**	**	**(–)**	**(–)**	**(–)**	**(–)**	**(–)**	#	**(–)**	**#**
13-(Z,E)-HODE/LA	(–)	(–)	(–)		(–)	(–)	(–)	(–)	(–)	(–)	(–)	*	(–)	(–)	(–)	(–)	(–)	(–)	(–)	(–)	(–)	(–)	(–)	#	(–)	(–)
13-(E,E)-HODE/LA	(–)	(–)	(–)		(–)	#	(–)	(–)	(–)	(–)	(–)	(–)	(–)	(–)	(–)	#	(–)	(–)	(–)	(–)	(–)	#	(–)	#	(–)	(–)
(9- and 13-(Z,E)-HODE)/LA ^a^	(–)	(–)	(–)		(–)	(–)	(–)	(–)	(–)	(–)	(–)	*	(–)	(–)	(–)	(–)	(–)	(–)	(–)	(–)	(–)	(–)	(–)	#	(–)	(–)
(9- and 13-(E,E)-HODE)/LA ^b^	(–)	(–)	(–)		(–)	(–)	(–)	(–)	(–)	(–)	(–)	(–)	(–)	(–)	(–)	(–)	(–)	(–)	(–)	(–)	(–)	(–)	(–)	(–)	(–)	(–)
a+b	(–)	(–)	(–)		(–)	(–)	(–)	(–)	(–)	(–)	(–)	*	(–)	(–)	(–)	(–)	(–)	(–)	(–)	(–)	(–)	(–)	(–)	#	(–)	(–)
7β-OHCh/total Ch	(–)	(–)	(–)		(–)	(–)	(–)	(–)	(–)	(–)	(–)	(–)	(–)	(–)	(–)	(–)	(–)	(–)	(–)	(–)	(–)	(–)	(–)	(–)	(–)	(–)
9-(Z,E)-HODE	(–)	*	**		(–)	**	(–)	(–)	(–)	*	(–)	(–)	(–)	**	(–)	**	(–)	(–)	(–)	**	(–)	**	(–)	(–)	(–)	(–)
9-(E,E)-HODE	(–)	**	(–)		(–)	(–)	(–)	(–)	#	**	(–)	(–)	(–)	(–)	(–)	(–)	(–)	(–)	(–)	(–)	(–)	(–)	(–)	(–)	(–)	(–)
10-(Z,E)-HODE	**	(–)	(–)		(–)	(–)	(–)	*	(–)	*	(–)	**	(–)	(–)	(–)	(–)	(–)	**	(–)	(–)	(–)	(–)	(–)	(–)	(–)	#
12-(Z,E)-HODE	(–)	(–)	(–)		(–)	(–)	(–)	(–)	(–)	(–)	(–)	(–)	*	(–)	(–)	(–)	(–)	(–)	**	(–)	(–)	(–)	(–)	(–)	(–)	(–)
**10- and 12-(Z,E)-HODE**	**	**(–)**	**(–)**		**(–)**	**(–)**	**(–)**	*	*	*	**(–)**	*	*	**(–)**	**(–)**	**(–)**	**(–)**	*	*	**(–)**	**(–)**	**(–)**	**(–)**	**(–)**	**(–)**	**#**
13-(Z,E)-HODE	(–)	(–)	**		(–)	**	(–)	(–)	(–)	(–)	*	(–)	(–)	**	(–)	**	(–)	(–)	(–)	**	(–)	**	(–)	(–)	(–)	(–)
13-(E,E)-HODE	(–)	(–)	(–)		(–)	(–)	(–)	(–)	##	(–)	(–)	(–)	(–)	(–)	(–)	(–)	(–)	(–)	(–)	(–)	(–)	(–)	(–)	(–)	(–)	(–)
(9- and 13-(Z,E)-HODE) ^c^	(–)	*	**		(–)	**	(–)	(–)	(–)	(–)	*	(–)	(–)	**	(–)	**	(–)	(–)	(–)	**	(–)	**	(–)	(–)	(–)	(–)
(9- and 13-(E,E)-HODE) ^d^	(–)	*	(–)		(–)	(–)	(–)	(–)	##	(–)	(–)	(–)	(–)	(–)	(–)	(–)	(–)	(–)	(–)	(–)	(–)	(–)	(–)	(–)	(–)	(–)
c+d	(–)	*	**		(–)	**	(–)	(–)	(–)	(–)	(–)	(–)	(–)	**	(–)	**	(–)	(–)	(–)	**	(–)	**	(–)	(–)	(–)	(–)
(Z,E)-HODE/(E,E)-HODE	(–)	(–)	(–)		(–)	**	(–)	(–)	**	(–)	(–)	(–)	(–)	(–)	(–)	**	(–)	(–)	(–)	(–)	(–)	**	(–)	(–)	(–)	(–)
5-HETE^e^	(–)	(–)	*		(–)	(–)	(–)	(–)	(–)	(–)	(–)	(–)	(–)	*	(–)	(–)	(–)	(–)	(–)	*	(–)	(–)	(–)	(–)	(–)	(–)
12-HETE^f^	(–)	(–)	*		(–)	(–)	(–)	(–)	(–)	(–)	(–)	(–)	(–)	*	(–)	(–)	(–)	(–)	(–)	*	(–)	(–)	(–)	(–)	(–)	(–)
15-HETE^g^	(–)	(–)	(–)		*	(–)	(–)	(–)	(–)	(–)	(–)	(–)	(–)	(–)	(–)	(–)	(–)	(–)	(–)	(–)	(–)	(–)	(–)	(–)	(–)	(–)
e+f+g	(–)	(–)	*		(–)	(–)	(–)	(–)	(–)	(–)	(–)	(–)	(–)	(–)	(–)	(–)	(–)	(–)	(–)	(–)	(–)	(–)	(–)	(–)	(–)	(–)
8-isoPGF2α	(–)	(–)	(–)		(–)	(–)	(–)	(–)	(–)	(–)	(–)	(–)	(–)	(–)	(–)	(–)	(–)	(–)	(–)	(–)	(–)	(–)	(–)	(–)	(–)	(–)
LA	#	*	**		(–)	**	(–)	(–)	(–)	(–)	**	#	(–)	**	(–)	**	(–)	(–)	(–)	**	(–)	**	(–)	*	#	(–)
totalCh	(–)	*	**		(–)	**	(–)	(–)	(–)	(–)	**	(–)	(–)	**	(–)	**	(–)	(–)	*	**	(–)	**	(–)	(–)	(–)	(–)
7β-OHCh	(–)	(–)	(–)		(–)	(–)	(–)	(–)	(–)	(–)	(–)	(–)	(–)	(–)	(–)	(–)	(–)	(–)	(–)	(–)	(–)	(–)	(–)	(–)	(–)	(–)

**Figure 5:**
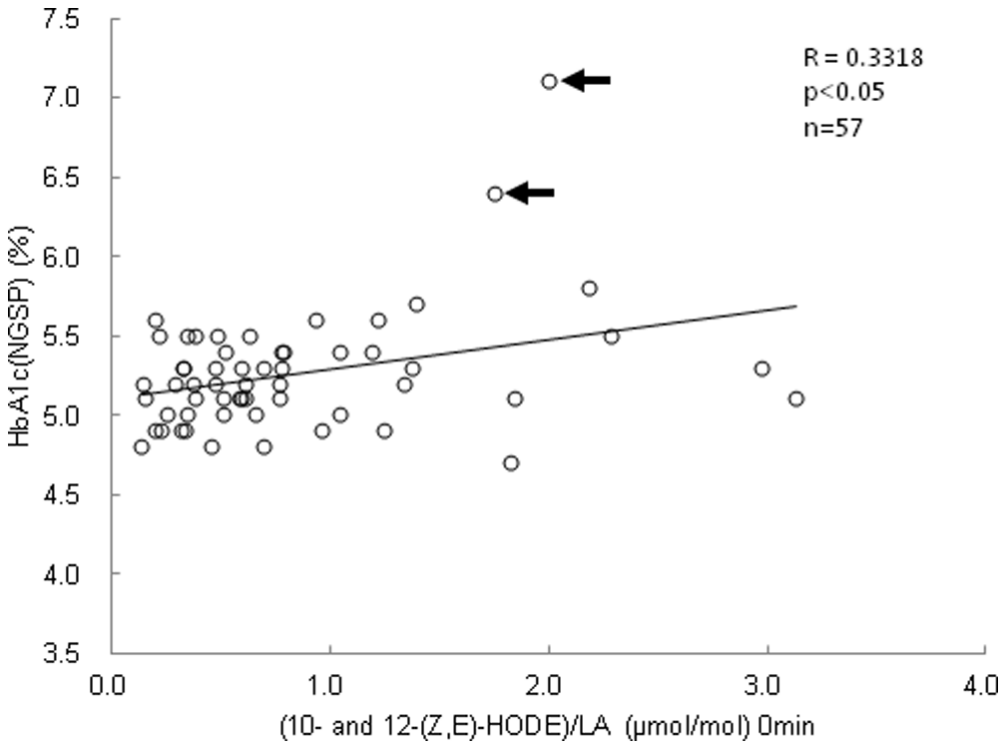
Correlation between HbA1c and fasting plasma levels of (10- and 12-(Z,E)-HODE)/LA. Allows indicates subjects in Group D.

* ^, #^, p<0.05;

** ^, ##^, p<0.01; (–), p>0.05 compared with the corresponding value in fasting lipid oxidation stress markers. Marks ^*, **^ and ^#, ##^ indicate positive and negative correlations, respectively. ND means not determined.Figure 5 shows the correlation between plasma levels of (10- and 12-(Z,E)-HODE)/LA and HbA1c in all subjects. Fasting plasma levels of (10- and 12-(Z,E)-HODE)/LA significantly increased with increasing levels of HbA1c (*p*<0.05, Fig. 5). Interestingly, the HbA1c levels of the 2 diabetes individuals (Group D, indicated by arrows) were much higher than the correlation line.

**Figure 6:**
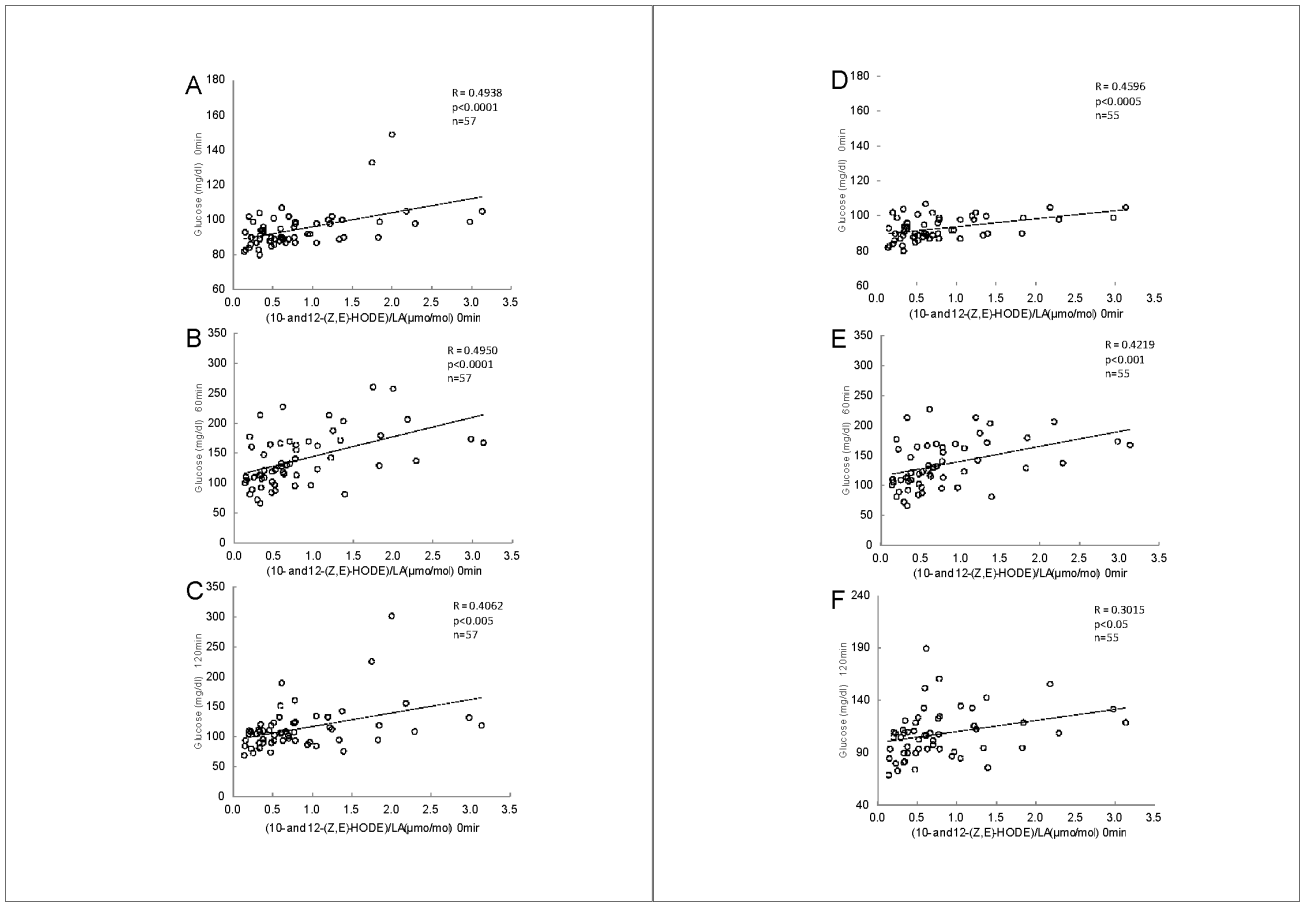
Correlation between fasting plasma levels of (10- and 12-(Z,E)-HODE)/LA in all subjects (A–C) and without diabetic type (D–F) and plasma glucose levels during OGTT. A and D, fasting glucose level; B and E, 60 min after the administration; C and F, 120 min after the administration.

Figure 6 shows the correlation between plasma levels of glucose and (10- and 12-(Z,E)-HODE)/LA in all subjects (A–C) and without diabetes (D–F). As shown, it is striking that fasting plasma levels of (10- and 12-(Z,E)-HODE)/LA were positively correlated with fasting glucose levels and glucose levels after the administration. Fasting plasma levels of 10- and12-(Z,E)-HODE showed the same tendency but did not exert stronger significance than (10- and12-(Z,E)-HODE)/LA (Table 2). Fasting plasma levels of LA were negatively correlated with fasting glucose levels and glucose levels after OGTT. Interestingly, there was no significant relationship between 9- and 13-(Z,E), 9- and 13-(E,E)-HODE (including those divided by LA), and fasting glucose levels and glucose levels after OGTT (Table 2). Furthermore, unexpectedly, HbA1c did not show a significant correlation with glucose levels in this study (Table 2). These results suggested that (10- and 12-(Z,E)-HODE)/LA may be potential biomarkers for prediction of glucose levels during OGTT.

**Figure 7:**
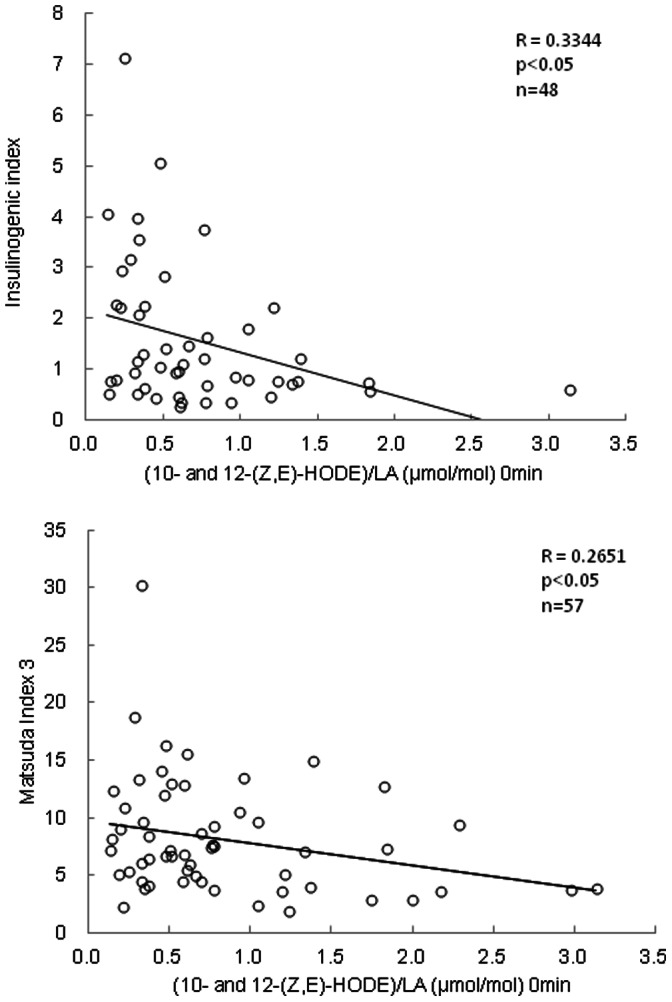
Correlation between fasting plasma levels of (10- and 12-(Z,E)-HODE)/LA in subjects without diabetic type and the insulinogenic index (A) or Matsuda Index 3 (B) during OGTT.

Figure 7 shows the correlations of the I.I. and Matsuda Index 3 against plasma levels of (10- and 12-(Z,E)-HODE)/LA in subjects without diabetes. Fasting plasma levels of (10- and 12-(Z,E)-HODE)/LA were negatively correlated with I.I. levels, a parameter of initial insulin secretion (*p*<0.05, Fig. 7A) and Matsuda Index 3 as an index of insulin resistance (*p*<0.05, Fig. 7B).

### Significance of Other Oxidative Stress Markers against Adipokines and Clinical Markers

The correlation between fasting levels of lipid oxidation stress markers and the levels of adipokines, clinical markers, insulin secretion index, and insulin resistance index determined during OGTT is summarized in Table 2. Both detecting pre-diabetes by medical examination and finding insulin abnormalities are important for early diagnosis of diabetes. Fasting plasma levels of 10-(Z,E)-HODE/LA, 12-(Z,E)-HODE/LA, and (10- and12-(Z,E)-HODE)/LA in all subjects (Table 2) were correlated to fasting glucose levels and glucose levels after OGTT. 9-(Z,E)-, 13-(Z,E)-, 9- and 13-(Z,E)-HODE; 9- and 13- (Z,E) and (E,E)-HODE; LA; and total cholesterol were significantly correlated with leptin and/or IL-6.

## Discussion

The present study was performed to elucidate the involvement of oxidative stress in the early stages of diabetes using several potential oxidative stress markers and to determine potential biomarkers for evaluating the early stages of diabetes. The involvement of oxidative stress in diabetes has been the subject of extensive studies. For example, glucose intolerance was associated with the high levels of thioredoxin as a marker of oxidative stress, correlated to the presence of coronary artery disease (CAD) [3]. NO metabolites, NO synthase inhibitors, thiols, and N-acetyl-β-glucosaminidase (NAGase) are biomarkers suitable for the detection of endothelial dysfunction and oxidative stress during the early stages of impaired response to insulin [6]. In addition, α-hydroxybutyrate is an early marker for both insulin resistance and impaired glucose regulation [7]. However, few studies have been conducted in normal subjects and have measured various biomarkers during OGTT.

We have recently developed a novel method to measure HODE from biological fluids and tissues; using this method, a considerable amount of the oxidation products of LA can be measured [23], [27], [29]. Reduction and saponification enabled us to measure hydroperoxides and hydroxides of both free and esterified forms of LA as the total HODE, which includes enzymatic and nonenzymatic products; 9- and 13-(Z,E)-HODE, nonenzymatic free radical-mediated products; 9- and 13-(E,E)-HODE, and specific nonenzymatic singlet oxygen-mediated products; 10- and 12-(Z,E)-HODE. Furthermore, we have recently reported that HODE levels in the plasma of healthy volunteers correlated significantly with corresponding oxidized LDL levels [25]. Thus, HODE is a useful biomarker for the assessment of oxidative status in humans.

The results of the present study clearly demonstrated that the plasma levels of (10- and 12-(Z,E)-HODE)/LA correlated significantly with several important clinical values for the diagnosis of diabetes, such as HbA1c, glucose, and insulin-related indices. Typical results of plasma glucose levels during OGTT were obtained in this study, i.e., every group except for Group D showed an increase in plasma glucose levels at 60 min and a decrease in plasma glucose levels at 120 min after the administration of 75 g glucose. It is interesting that the levels of lipid peroxidation markers examined in this study did not increase with increasing levels of glucose, suggesting that they can reflect the long-term status. The reason why (10- and 12-(Z,E)-HODE)/LA in Group N decreased during OGTT is not known at present, but metabolism and excretion of oxidized lipids could be a part of the reason.

Plasma levels of malondialdehyde and erythrocyte superoxide dismutase, as markers of oxidative stress, were significantly elevated in elderly subjects with type 2 diabetes mellitus [2]. This was thought to be due to increased free radical production possibly resulting from hyperglycemia. Further, (E,E)-HODE were derived specifically from free radical-mediated oxidation and their plasma levels were 10 times higher than those of 10- and 12-(Z,E)-HODE. Accordingly, it had been postulated that (E,E)-HODE may be a prominent biomarker for the early detection of diabetes. It is surprising that fasting levels of (10- and 12-(Z,E)-HODE)/LA, but not (E,E)-HODE, showed significant correlations with fasting and OGTT (60, 120 min) glucose levels in individuals (Fig. 6 and Table 2). Furthermore, the levels of (10- and 12-(Z,E)-HODE)/LA correlated with HbA1c, which are criteria for assessing long-term plasma glucose control (Fig. 5). Importantly, there were 9 normal type, 3 out of 7 high-normal-type, 2 out of 5 IGT, and 2 diabetic type individuals whose fasting levels of (10- and 12-(Z,E)-HODE)/LA were more than 1.0. It is noteworthy that 5 out of 9 normal type individuals, whose (10- and 12-(Z,E)-HODE)/LA were more than 1.0, showed high levels (>140 mg/dL) of glucose 60 min after administration during OGTT, who might lead to high-normal type and IGT in future, although detailed cohort studies are needed.

Fasting levels of (10- and 12-(Z,E)-HODE)/LA increased with decreasing I.I. and Matsuda Index 3 scores, suggesting that (10- and 12-(Z,E)-HODE)/LA reflected the oxidative status correlated to insulin secretion and resistance (Fig. 7). It is widely accepted that lower insulin secretion and deterioration of insulin resistance lead to a vicious cycle of glucose toxicity for type 2 diabetes, which involves oxidative stress [35], [36]. Insulin gene expression is suppressed by oxidative stress in β-cells when hyperglycemia results in lowering insulin secretion [37], [38]. Therefore, the data presented in this study suggested that (10- and 12-(Z,E)-HODE)/LA reflected insulin secretion from pancreatic β-cells and insulin resistance and then correlated very well with plasma levels of glucose during OGTT.

Singlet oxygen oxidizes lipids by nonradical and nonenzymatic mechanisms [39]. The oxidation of linoleates by singlet oxygen gives 9-, 10-, 12-, and 13-(Z,E)-HPODE [40]. Since free radical-mediated oxidation of linoleates yields 9- and 13-(Z,E)-HPODE, 10- and 12-(Z,E)-HPODE is a specific product of singlet oxygen oxidation. Oxidation by singlet oxygen *in vivo* has been widely studied from the viewpoint of photodynamic therapy or deleterious damage, such as in porphyria of the skin. Singlet oxygen is produced *in vivo* by the activation of neutrophils [41], [42], including the reaction of hydrogen peroxide with hypochlorite, which produced by myeloperoxidase (MPO), a heme protein secreted by activated phagocytes, by eosinophils through peroxidase-catalyzed mechanism [43], and by bimolecular interactions of lipid peroxyl radicals [44]. This study is the first report showing 10- and 12-(Z,E)-HODE but not 9- and 13-HODE highly correlated with clinical values for diabetes. It is speculated that neutrophils which contains MPO is recruited by hyperglycemia to adipose cells or β-cells to form singlet oxygen. Further studies are needed, especially on a molecular level, to determine the mechanisms of 10- and 12-(Z,E)-HODE formation.

The markers measured in this study, leptin, adiponectin, IL-6, TNF-α, and hs-CRP, showed no correlation with 10- and 12-(Z,E)-HODE. These adipokines can be also biomarkers for diagnosis of diabetes, but not for early stages of the disease. Indeed, the plasma levels of these adipokines in normal, high-normal, and pre-diabetes individuals were within the normal reference levels.

In conclusion, the results of the present study clearly showed that fasting oxidative stress markers (10- and 12-(Z,E)-HODE)/LA were positively correlated with fasting glucose plasma levels and plasma glucose levels after glucose administration during OGTT. Furthermore, these markers reflected the insulin secretion and resistance index. These findings clearly indicated that singlet oxygen was indeed involved in the early stages of diabetes. More importantly, these lipid peroxidation products are sensitive to dysfunction during normal conditions. Thus, it can be concluded that (10- and 12-(Z,E)-HODE)/LA are suitable biomarkers for the evaluation of the early stages of diabetes.
